# Medication control of flunixin in racing horses: Possible detection times using Monte Carlo simulations

**DOI:** 10.1111/evj.13532

**Published:** 2021-11-25

**Authors:** Taisuke Kuroda, Yohei Minamijima, Motoi Nomura, Shozo Yamashita, Masayuki Yamada, Shunichi Nagata, Hiroshi Mita, Norihisa Tamura, Kentaro Fukuda, Atsutoshi Kuwano, Kanichi Kusano, Pierre‐Louis Toutain, Fumio Sato

**Affiliations:** ^1^ Clinical Veterinary Medicine Division Equine Research Institute Japan Racing Association Shimotsuke Japan; ^2^ Drug Analysis Department Laboratory of Racing Chemistry Utsunomiya Japan; ^3^ Equine Department Main office Japan Racing Association Minato‐ku Japan; ^4^ Comparative Biomedical Sciences The Royal Veterinary College London UK; ^5^ Intheres Ecole Nationale Vétérinaire de Toulouse Toulouse France

**Keywords:** doping, horse, irrelevant plasma concentration, irrelevant urine concentration, medication control

## Abstract

**Background:**

For medication control in several jurisdictions, withdrawal time is the period of refrain from racing after drug administration. It is set by adding a safety period to an experimental detection time. However, there are no reports of statistical analyses of detection time for the determination of withdrawal time in flunixin meglumine‐treated horses.

**Objective:**

To analyse the population pharmacokinetics of flunixin in horses through the generation of a dataset for detection time statistical analysis and predictions via Monte Carlo simulation.

**Study design:**

Experimental study.

**Methods:**

Drug plasma and urine concentrations following single intravenous administration of flunixin 1.1 mg/kg bodyweight (BW) in 10 horses and multiple administration of q 24 hours for 5 days in 10 horses were measured using liquid chromatography with tandem mass spectrometry (LC‐MS/MS). Data were modelled using a nonlinear mixed effect model followed by Monte Carlo simulation. Irrelevant plasma concentration (IPC) and irrelevant urine concentration (IUC) were calculated using the Toutain approach. Detection times were obtained considering the time after the last administration for selected quantiles of 5000 hypothetical horses under the international screening limit (ISL) proposed by the International Federation of Horseracing Authorities (plasma: 1 ng/mL, urine; 100 ng/mL).

**Results:**

For a regimen of 1.1 mg/kg BW q 24 hours, the IPC and IUC values were 2.0 and 73.0 ng/mL respectively. Detection times in plasma above the ISL for 90% of simulated horses were estimated as 74 hours after a single 1.1 mg/kg dose administration, 149 and 199 hours after multiple doses over 5 days at either 24‐ or 12‐hour intervals respectively. Corresponding detection times in urine were 46, 68 and 104 hours respectively.

**Main limitation:**

Only female horses were investigated.

**Conclusions:**

Statistical detection times for different flunixin meglumine regimens indicated a delay of detection time in plasma after multiple administrations under ISL.

## 1 INTRODUCTION

Flunixin meglumine is a one of the most commonly employed NSAIDs in cases of inflammation and pain associated with soft tissue conditions in horses.^1^, ^2^, ^3^, ^4^ It is also considered efficacious in controlling abdominal pain, and thus is the standard therapy for equine colic.^5^, ^6^, ^7^

Most horse racing regulatory authorities have distinct frameworks for doping control and medication control. In the case of doping drugs, such as anabolic steroids, the objective is to detect any trace through the most sensitive analytical methods.^8^, ^9^, ^10^ For medication control of drugs like flunixin meglumine, an irrelevant plasma concentration (IPC) and irrelevant urine concentration (IUC) are estimated in order to guarantee fair competition in parallel to proper veterinary care. The IUC and IPC are determined via the Toutain model approach based on PK/PD analysis of available data (efficacious dose and corresponding plasma and urine disposition), as per the recommendation of the European Horserace Scientific Liaison Committee (EHSLC).^10^

European Horserace Scientific Liaison Committee defines detection time as the interval between the time of the last administration and the time at which the observed urine (plasma) concentrations are below the screening limit (SL).^11^ Detection time experiments are usually conducted after the administration of a single dose in six to eight horses. However, detection time is only preliminary information provided without any statistical basis. As described by the EHSLC or the Fédération Equestre Internationale, detection time depends on various factors, including the dosage regimen, route of administration, pharmaceutical formulation, breed, age, sex and, most importantly, the number of investigated horses. The last factor has been explored through a series of Monte Carlo simulation studies in order to allow prescribers to set a withdrawal time for a particular horse, including an additional safety period which will be even longer than detection times observed for a limited number of horses.^11^ Although the pharmacokinetics of flunixin in horses have already been established,^12^, ^13^, ^14^, ^15^ detection times have not been subjected to advanced statistical analysis. Additionally, detection times after multiple clinical administrations have not been reported in the scientific literature or by any organisation. Thus, we carried out the statistical analysis of detection time to predict its values for different regimen, including multiple administrations, via Monte Carlo simulation for the purpose of medication control.

## 2 MATERIALS AND METHODS

### 2.1 Study design

Twenty healthy 3‐ to 10‐year‐old experimental Thoroughbred female horses with a bodyweight (BW) of 428‐530 kg were used. Horses were examined by a veterinarian and found healthy prior to the investigation. Horses were kept in individual stalls during experiments and had ad libitum access to grass, hay and water. Straw was completely replaced every day.

Flunixin meglumine dose (1.1 mg/kg BW flunixin) was determined based on a previous report.^13^ Flunixin meglumine was administered into the right jugular vein via a short bolus infusion (<10 seconds). Ten horses received a single administration of flunixin (Banamine 5% injection; DS Pharma Animal Health Co. Ltd.), and blood samples were collected at 0 (prior to administration) as well as 0.5, 1, 3, 6, 9, 24, 30, 48, 72, 120, 144, 168, 192, 216 and 240 hours after administration. The other 10 horses were subjected to a 5‐day regimen of q 24 hours. Flunixin was administered at 0, 24, 48, 72, 96 hours, and blood samples were collected at 24, 48, 72, 96 (prior to the next administration) as well as at 96.5, 97, 99, 102, 105, 120, 126, 144, 168, 192, 216, 240, 264, 288 312 and 336 hours after the first administration. Blood samples (10 mL) were taken from the left jugular vein using a 16 G catheter (Becton Dickinson Company) and collected in heparinised vacuum blood collection tubes (Terumo). Urine was collected through a urine catheter (Becton Dickinson Company) close to scheduled time points at 3, 6, 9, 24, 30, 48, 72, 96, 120 hours after the single administration or at 99, 102, 105, 120, 126, 144, 168, 192, 216, 240, 264, 288, 312, 336 hours after the first administration. The plasma was immediately centrifuged at 1500 g for 10 minutes. Plasma and urine samples were stored at −20°C until analysis.

### 2.2 Sample analysis

Twenty microlitres of methanol (containing 1 μg/mL diclofenac‐d4 [Toronto Research Chemicals] as an internal standard) and 0.1 mL 1 mol/L sodium hydroxide were added to 0.1 mL plasma or urine, and the mixture was incubated at room temperature for 10 min. To hydrolyse urine, 1 mL 1 mol/L acetate buffer (pH 5.0) and 4 mL tert‐butyl methyl ether were added, and the mixture was stirred for 5 minutes. The upper organic phase was dried under a nitrogen stream at 40°C. The residue was reconstituted with 0.5 mL of 0.1%(v/v) formic acid containing water and acetonitrile (9:1). For LC/MS/MS analysis, 5 μL of each sample was injected into the system described below.

Plasma and urine flunixin concentrations were measured using an LC‐MS/MS system consisting of a mass spectrometer (Sciex 4500QTRAP; SCIEX Corporation) equipped with a liquid chromatography system (Nexera X2 system; Shimadzu Corporation). UPLC separation was performed on an ACQUITY UPLC BEH (100 mm, 2.1 mm, 1.7 μm) column (Waters Corporation) with a mixture of 0.1% formic acid and acetonitrile as the mobile phase. Mass spectrometry parameters were optimised and set as follows: negative electron ionisation at a spray voltage of −3500 V, a capillary temperature of 600°C and a nebuliser gas pressure of 60 psi. Quality control samples for calibration were prepared by adding standard flunixin (Fujifilm Wako Pure Chemical Corporation) to blank horse plasma or urine. Flunixin calibration curves were validated over 0.1‐100 ng/mL for plasma and 3‐3000 ng/mL for urine. The best linear fit and regression were obtained with a 1/*y* ^2^ weighing factor. The calculated correlation factors (*R* ^2^) for linearity were always greater than 0.995. Intra‐ and inter‐day accuracy and precision were assessed using QC samples at 0.1, 0.2, 3.0 and 80 ng/mL for plasma and 3, 6, 100 and 2400 ng/mL for urine. Intra‐assay and inter‐assay precision were within 7.24% and 10.1%, while intra‐assay and inter‐assay accuracy were within 8.80% and 11.2% for plasma and urine respectively. To evaluate dilution integrity, flunixin was spiked at concentrations of 1000 ng/mL in plasma and 50 000 ng/mL in urine. Plasma and urine were diluted with blank plasma or urine to reach final concentrations of 10 and 100 ng/mL respectively. Diluted samples were analysed using calibration standards, with accuracy and precision within 15.0%. Quantifications were performed via selected ion monitoring, with ion transitions of m/z 294.7‐208.8 for flunixin and m/z 298.0‐217.0 for diclofenac‐d4.

### 2.3 Pharmacokinetic analysis

Plasma pharmacokinetic analyses were conducted using a nonlinear mixed effect (NLME) model on commercially available software (Phoenix WinNonlin version 8.3, Certara). A three‐compartment structural model was selected based on the likelihood ratio test and the Akaike information criterion. The model parameters were the central (V1) and two peripheral (V2, V3) volumes of distribution, plasma clearance (CL) and the inter‐compartmental distribution clearances (CL2, CL3).

The plasma flunixin concentration is the main determinant of urinary concentration ensuring an a priori parallelism of flunixin decay in plasma and urine. Accordingly, after flunixin administration, the steady‐state urine‐to‐plasma ratio Rss (U/P) of flunixin concentration was estimated via the addition of an equation expressing the urinary concentration as proportional to that in plasma. Rss, the factor of proportionality, was obtained from the best fit of both plasma and urinary concentrations. Only urinary data collected at 24 hours post administration were considered in order to ensure pseudodistribution equilibrium, supporting plasma and urinary concentration parallelism.

The statistical approach describing the inter‐animal variability was included in the population model. The interindividual variability for a given parameter was described using an exponential model:

(1) $$\theta_{\text{parameter}\_i}=\theta_{\text{tv}\_\text{parameter}}\cdot \exp\left(\eta_{i}\right),$$

where *θ*_parameter_i_ is the value of theta for a given parameter in the *i*th animal, *θ*tv_parameter is the typical population value of parameters and *η*_i_ (etai) is the deviation associated with the *i*th animal from the corresponding theta population value. An exponential model was selected because the estimated theta parameters must be positive, and their distributions are generally right‐skewed. Thus, variability between horses was estimated from their individual etas. The distribution of etas was assumed normal with a mean of 0 and a variance *ω* ^2^.

To report interindividual variability as a coefficient of variation, Equation (2) was used for the conversion of variance terms (*ω* ^2^) into a coefficient of variation (CV%).

(2) $$\text{CV}\left(\%\right)=100\times \sqrt{\exp(\omega^{2})‐1.}$$

Shrinkage of the random effects (eta) towards the means was described as:

(3) $$\text{Shrinkage}=1‐\frac{\text{var}(\eta_{r})}{\omega^{2}},$$

where var(*η*_r_) is the variance of Empirical Bayes (“post hoc”) estimates (EBEs) of *η*_s_. When the shrinkage for eta was >0.3, it was considered that the data would not allow for the robust estimation of this random component. Since there were no parameters associated with a shrinkage for etas >0.3, a random component was added for all parameters. The residual error model was an additive plus a multiplicative (proportional) model of the form.

(4) $$C\left(t\right)=f\left(\theta ,\;\text{Time}\right)\times \left(1+\varepsilon_{1}\right)+\varepsilon_{2},$$

with *ε*1, the multiplicative error term, having a mean of 0 and a variance noted *σ*1

$$\varepsilon 1\approx N(0,\sigma 1^{2}),$$

and *ε*2, the additive error term, having a mean of 0 and a variance noted *σ*2

$$\varepsilon 2\approx N(0,\sigma 2^{2}).$$

The additive sigma was reported as its standard deviation noted with the same units as plasma concentration (µg/mL), and the multiplicative sigma was reported as a coefficient of variation. The precision of the parameters was estimated using a bootstrap tool (*n* = 50 replicates).

Plasma clearance was used to calculate the effective plasma concentration (EPC) via Equation (5).^10^

(5) $$\text{EPC}=\frac{\text{Dose}\;\text{per}\;24\;h}{\text{Clearance}\;\text{per}\;24\;h}.$$

A dose of 1.1 mg/kg per 24 hours and clearance estimated by the model were used to compute the EPC. The IPC was obtained by dividing the EPC by the selected uncertainty factor (500), and the IUC was obtained by multiplying flunixin IPC by the corresponding Rss estimated via the model.^10^

Monte Carlo simulation was used to generate plasma and urine concentrations of a virtual population of 5000 horses using individual predictions (IPRED) (eta was as estimated), corresponding to 1.1 mg/kg single and multiple (q 12 h and q 24h for 5 days respectively) administrations. Using this meta‐population, the detection time corresponding to the time of last administration under the International Federation of Horseracing Authorities (IFHA) international screening limit (ISL) in plasma (1 ng/mL) and urine (100 ng/mL) was estimated.^16^ These data were then analysed with the Phoenix statistical tool in order to compute the quantiles of interest (5, 10, 25, 50, 75 90, 95th).

In the US, the Racing Medication and Testing Consortium (RMTC) compute withdrawal time with its confidence interval as done for drug residues in food‐producing animals.^17^ The RMTC SL for plasma flunixin is 5 ng/mL. We also generated a meta‐population of 5000 detection times for this US SL in addition to computing the 95% confidence interval of the 95th percentile considered by the RMTC via the nonparametric bootstrap tool of Crystal Ball (Oracle Corporation), which is appropriate for estimating the reliability of forecast statistics.

## 3 RESULTS

**FIGURE 1:**
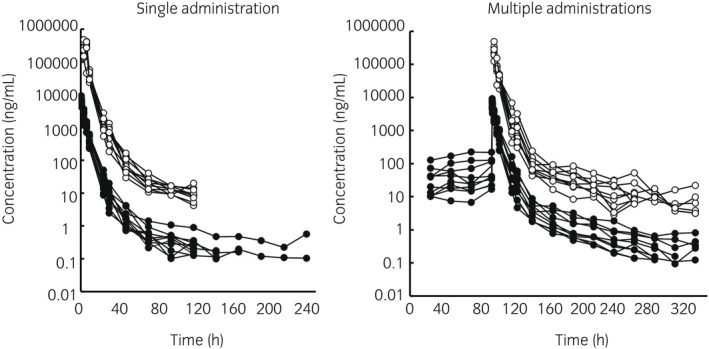
Semilogarithmic spaghetti plots of flunixin disposition curves in plasma and urine after single‐dose administration of 1.1 mg/kg BW flunixin in 10 horses and q 24 h multiple administrations in 10 horses. Solid circles indicate flunixin concentrations in plasma, open circles indicate flunixin concentrations in urine

**FIGURE 2:**
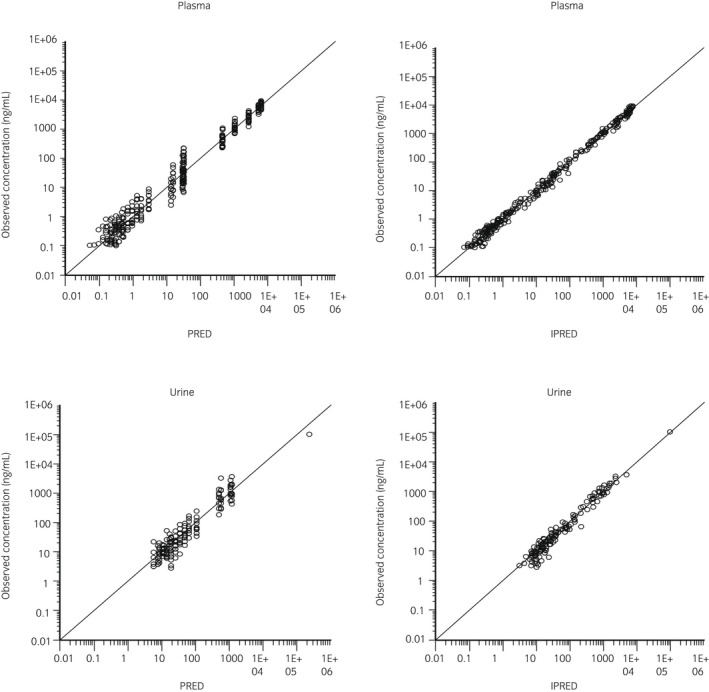
Logarithmic plots of observed flunixin concentrations in plasma (top) and urine (bottom) vs population (PRED) (left plots) and individual predictions (IPRED) (right plots)

**FIGURE 3:**
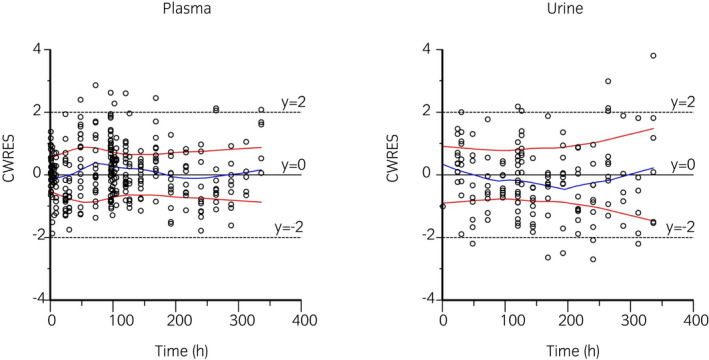
Conditional weighted residuals (CWRES) vs time plot for plasma (left) and urine (right). Values of CWRES should be approximately N (0, 1) and hence concentrated between *y* = −2 and *y* = +2. Inspection of the figure indicates that data were evenly distributed about zero and that the trends (as given by the blue line and the red line with its negative reflection) did not show any fanning, thus indicating no bias in the structural model

**FIGURE 4:**
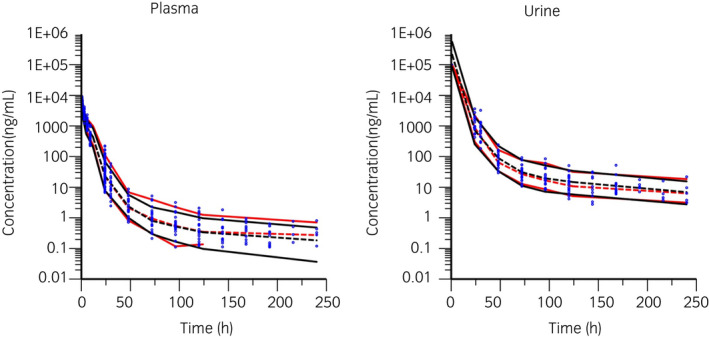
Visual predictive check of observations vs time after dose in plasma (left) and urine (right) in the case of a single dose of 1.1 mg/kg BW and 1.1 mg/kg BW q 24 h multiple administration. The observed and predicted 10th and 90th percentiles are indicated via solid red and black lines respectively. The observed and predicted 50th percentiles (median) are indicated via red and black broken lines respectively. Blue dots represent individual raw data

Semilogarithmic plots of the disposition curves for plasma and urine flunixin concentrations in each horse are depicted in Figure 1. Descriptive statistics characterising flunixin elimination in plasma and urine are given in Table 1. The last detection time of plasma concentration above the ISL (1 ng/mL) was 30‐96 and 48‐144 hours after single and multiple flunixin meglumine administration respectively. The last detection time of urine concentration above the ISL (100 ng/mL) was 30‐48 hours after single and multiple administration. Logarithmic plots of the observed drug plasma and urine concentrations vs population predictions (PRED) and IPRED are shown in Figure 2. Data were evenly distributed about the line of identity, indicating no major bias in the model's population component. The plot of conditional weighted residuals vs time indicated that residuals were randomly scattered around zero with no systematic trend, supporting residual error model selection (Figure 3). Visual Predictive Check ensured that simulated data were consistent with observed data (Figure 4).

**TABLE 1:** Descriptive statistics for the plasma and urine disposition of flunixin after single and multiple intravenous administration of 1.1/kg BW in 20 horses

	Unit	Plasma	Urine
Single administration (range)			
*C*_max_	µg/mL	None	270.5‐419.5
*C*_0_	µg/mL	6.54‐9.91	None
*T*_max_	h	None	3‐6
Last detection time above a concentration of 1 ng/mL	h	30‐96	None
Last detection time above a concentration of 100 ng/mL	h	9	30‐48
Multiple administration (range)			
*C*_max_	µg/mL	None	125.0‐486.0
*T*_max_ after last administration	h	None	0.5
Last detection time after last administration for a concentration of 1 ng/mL	h	48‐144	None
Last detection time after last administration for a concentration of 100 ng/mL	h	9	30‐48

Abbreviations: *C*_0_, initial concentration; *C*_max_, maximal concentration; *T*_max_, time for *C*_max_.

The interindividual variability computed with Equation (2) and post‐hoc values are given in Table 2. The variability for Rss was highest, with a coefficient of variation of 55.5% corresponding to Rss post‐hoc values from 6.0 to 97.1. Bootstrap estimates of the primary structural parameters (thetas), secondary parameters and their associated coefficients of variation, as a measure of the precision of their estimation, are given in Table 3. The bootstrap median estimates were 0.046 L/kg/h for clearance, 0.196 L/kg for steady‐state volume of distribution and 37.1 for Rss. An EPC of 996 ng/mL was computed after 1.1 mg/kg BW q 24 hours. The flunixin IPC and IUC were estimated at 2.0 and 73.0 ng/mL respectively (Table 4). Using the meta‐population of 5000 horses, detection times to the ISL of IFHA for 90% of simulated horses were 74, 112, 149 and 199 hours for plasma after 1.1 mg/kg single, q 12 hours 1 day, q 24 hours and q 12 hours 5‐day administration and 46, 61, 68 and 104 hours for urine (Table 5). It was estimated that withdrawal times corresponding to the plasma RTMC SL of 5 ng/mL for 95% of simulated horses with a statistical (unilateral) confidence of 5% as 49, 53, 58 and 90 hours after 1.1 mg/kg single, q 12 hours 1 day, q 24 hours and q 12 hours 5‐day administration respectively.

**TABLE 2:** The interindividual variability (CV%) and range of individual post‐hoc values of primary parameters for flunixin in horses as obtained via a three‐compartment model

Primary structural parameters	Units	Typical value (single run)	CV%	Range of post‐hoc values
tvV	L/kg	0.149	2.0	0.119–0.182
tvV2	L/kg	0.012	4.2	0.007–0.028
tvV3	L/kg	0.029	19.1	0.016–0.050
tvCL	L/kg/h	0.047	30.0	0.029–0.066
tvCL2	L/kg/h	0.00015	14.8	0.00008–0.00040
tvCL3	L/kg/h	0.00443	10.3	0.00183–0.01369
Rss		36.8	55.5	6.0–97.1

Abbreviations: CL, plasma clearance; CL2, CL3, distribution clearances; Rss, steady‐state urine‐to‐plasma ratio; Tv, typical value; V1, volume of distribution of the central compartment; V2, V3, the volume of distribution of the peripheral compartments.

**TABLE 3:** Bootstrap estimates of population primary parameters for flunixin in horses with a three‐compartment model (median, CV%, 2.5% and 97.5% percentiles)

	Units	Typical values (Median)	CV%	2.50%	97.50%
Primary structural parameters					
tvV	L/kg	0.151	5.8	0.137	0.173
tvV2	L/kg	0.013	9.8	0.010	0.014
tvV3	L/kg	0.03	8.9	0.023	0.033
tvCL	L/kg/h	0.05	7.0	0.04	0.05
tvCL2	L/kg/h	0.00015	7.7	0.00013	0.00017
tvCL3	L/kg/h	0.00429	18.2	0.00312	0.00687
Rss		37.1	14.5	25.0	46.2
tvCMultStdev (residual, proportional)	Scalar	0.30	8.0	0.25	0.34
stdev0 (residual, additive)	ng/L	0.063	27.8	0.040	0.099
stdev1 (residual, additive)	ng/L	0.080	8.2	0.079	0.093
Secondary parameters					
Half‐life alpha	h	2.0	4.7	1.7	2.1
Half‐life beta	h	5.1	6.9	4.4	5.9
Half‐life gamma	h	58.8	7.7	49.4	65.9
Vss	L/kg	0.191	4.9	0.177	0.210
MRT (IV)	h	4.2	5.1	3.9	4.5

Abbreviations: CL, plasma clearance; CL2, CL3, distribution clearances; CMultStdev, proportional component of residual error; MRT, mean residence time; stdev0, additive component of the residual; tv, typical value; V1, volume of distribution of central compartment; V2, V3, volume of distribution of peripheral compartments; Vss, steady‐state volume of distribution.

**TABLE 4:** Effective and irrelevant flunixin concentrations

Variables	Units	Estimates	Precision (CV%)
EPC	ng/mL	995.9	6.9
IPC	ng/mL	2.0	
IUC	ng/mL	73.0	

Abbreviations: EPC, effective plasma concentration; IPC, irrelevant plasma concentration; IUC, irrelevant urine concentrations.

**TABLE 5:** Detection times (h): quantiles (5, 10, 25, 50, 75, 90 and 95th) for a hypothetical horse population obtained via Monte Carlo simulation for the international screening limits of flunixin in plasma and urine

Quantiles	Plasma (international screening limit: 1 ng/mL)				Urine (international screening limit: 100 ng/mL)			
	Dosage regime (1.1 mg/kg)							
	Single	q 12 h 1 d	q 24 h 5 d	q 12 h 5 d	Single	q 12 h 1 d	q 24 h 5 d	q 12 h 5 d
5	47	53	58	76	30	37	38	45
10	49	55	62	84	32	40	41	48
25	52	60	70	107	35	44	45	56
50	57	68	91	139	39	49	51	68
75	64	87	121	170	42	55	59	84
90	74	112	149	199	46	61	68	104
95	84	128	164	217	49	65	75	117

## 4 DISCUSSION

The pharmacokinetics of flunixin were previously reported in several studies,^13^, ^14^, ^18^, ^19^ but its population pharmacokinetics in horses remain to be established. The average steady‐state volume of distribution and clearance reported were 0.137‐0.157 L/kg and 0.046‐0.062 L/kg/h, respectively, similar to our results.^13^, ^14^ The numerical value of the Rss is rarely mentioned in the literature, but the average Rss value obtained herein (Rss = 37) seems consistent with those deduced from published raw data or corresponding flunixin depletion curves.^13^ For convenience of urine collection, only female horses were used in this study. We believe that this has no impact on the generality of our results as no sex differences in flunixin disposition have been reported. Flunixin clearance was significantly lower in old compared with young horses.^12^ To prevent possible age‐related bias, we selected equids representative of the racehorse population.

With major advances in analytical techniques, minute drug levels can be detected on racing day. Hence, an SL based on IPC and IUC should be estimated in order to control competition fairness without impeding proper veterinary care.^9^, ^10^ IPC and IUC were calculated from the EPC divided by a default safety factor of 500. This scaling factor of 500 is the product of 50 and 10. The factor of 50 is to transform a putative average EPC of 50% (the EC50) predicted by a PK/PD *E*_max_ model into the corresponding average residual 2% effective concentration considered clinically insignificant. The factor of 10 reflects the interindividual variability for the PK (3.3) and PD (3.3) components and ensures equity between horses.^20^, ^21^, ^22^ Therefore, it is assumed that the EPC should be close to the EC50. In the PK/PD analysis of the clinical effect of stride length and rest angle in experimental arthritis, the EC50 of flunixin was reported to range from 0.24 to 0.93 µg/mL, which was close to the EPC computed herein (0.96 µg/mL).^23^ The IPC and IUC calculated in this study were similar to the ISL of IFHA.

Several studies have explored flunixin's effects on thromboxane B2 production in horses, as thromboxane generation is considered an index of NSAID efficacy.^13^, ^23^, ^24^, ^25^ A single 1.1 mg IV flunixin administration maintained a significant effect for 24 hours, which was lost when the plasma concentration decreased to 10‐20 ng/mL.^13^, ^25^ Considering a PK/PD factor of 50, the average IPC can be estimated to 18.9 ng/mL, which is close to the concentration at which the significant effect on thromboxane production was compromised, indicating the validity of EPC and IPC.

Detection time experiments are often conducted after a single dose. It should be considered that detection time depends on various biological and clinical factors. These include between‐subject variability of PK parameters, and differences in administration route, dosage or formulation. It has already been reported that multiple doses may prolong detection time.^26^, ^27^ In this study, 10 horses were administered flunixin q 24 hours for 5 days, and data were subjected to Monte Carlo simulation. Veterinarians can select the withdrawal time based on the results provided in Table 5.

European Horserace Scientific Liaison Committee and IFHA indicated 144 hours as the detection time.^28^, ^29^ Comparison of Monte Carlo simulation‐generated detection times with these previously published detection times revealed that hypothetical urine detection times were below 144 hours for all dosage regimens. Thus, a positive urine test is unlikely even in multiple administration after 144 hours from the last administration. Further, 95% of the 5000 population were below the SL of plasma after a single and q 12 hours 1‐day flunixin administration. However, the plasma SL would be exceeded after multiple dosing over 5 days. In order to avoid positive cases, regulatory authorities testing plasma with reference to IFHA’s plasma ISL should inform prescribing veterinarians that detection times may be prolonged in the case of multiple flunixin meglumine dosing. A word of caution must be expressed regarding the risk of flunixin oral recycling. Seventy‐five per cent of the intravenously administered flunixin is excreted via urine.^30^ Such an amount can be partly recycled when the horse ingests litter straw contaminated with its own urine as reported for flunixin and meclofenamic acid.^31^, ^32^, ^33^ Thus, we replaced the straw in stalls daily, preventing a prolonged detection time due to flunixin recycling.

When considering the IFAH ISL, detection times in plasma were longer than in urine. This is related to the Rss difference used by the IFHA when determining the plasma and urine ISL (ie 100) and the one computed in this study (ie 37). Of note, the urine‐to‐plasma ratio is subject to considerable variation. In our horses, the interindividual variability of Rss was 55.5%, with values ranging from 6 to 97. The urine‐to‐plasma ratio depends on various factors, such as diet and water intake, rendering urine less attractive for robust medication control.^10^

The alternative to the EHSLC approach followed herein is that of RMTC in the USA, which consists of calculating statistical withdrawal times as for drug residues in food‐producing animals. This method guarantees that a positive result to be highly unlikely. Its disadvantage is the need for higher SLs as a ‘price’ of the certainty of detection time/withdrawal time as stakeholder priorities. However, this would not result in calculating long withdrawal times unacceptable for stakeholders. Beyond technical considerations, these two approaches reflect an inversion of animal welfare and the certainty of detection time/withdrawal time as stakeholder priorities. RMTC indicated an SL of flunixin at 5 ng/mL for plasma with a corresponding withdrawal time of 144 hours for a single‐dose administration at 1.1 mg/kg via the IV route.^34^ We computed withdrawal times according to RMTC with the 95/95 tolerance interval using our meta‐population of 5000 horses. For all scenarios, the withdrawal times were below 144 hours, including under multiple‐dose administration. It is interesting to observe that despite statistical protection, withdrawal times calculated according to the method of the RMTC were clearly shorter than the corresponding detection time obtained via the EHSLC method. This is explained not only by different SLs (5 vs 1 ng/mL) but also by the fact that flunixin plasma depletion obeys a three‐compartment model and, for our investigated horses, the SL of 5 ng/mL was located in the second phase of the depletion curve (half‐life of about 5 hours), while the SL of 1 ng/mL was located in the very late terminal phase (half‐life of about 59 hours).

In the formal context of a Risk Analysis for medication control as described by Toutain,^9^ the first step is the Risk Assessment, identical between EU and US approaches and requiring the generation of robust data to be analysed via the advanced scientific tools, such as population modelling.^9^ It is only during the second step, Risk Management, that the same data and results can be handled differently, leading to different recommendations based on the values specific to each jurisdiction. Within this framework of Risk Assessment, we generated data on flunixin meglumine which could be utilised in the approaches described above.

In conclusion, the flunixin IPC and IUC calculated for 20 horses were consistent with the IFHA ISL. MCS indicated that a detection time of 144 hours, as proposed by EHSLC and IFAH, is appropriate for a single flunixin meglumine administration at the assessed dose. However, the delay after multiple administrations may not be sufficient to ensure negativity, especially in plasma. This study provides statistical detection times which should facilitate the determination of individual withdrawal times by clinicians.

## ETHICAL ANIMAL RESEARCH

All experiments were approved by the Animal Care and Use Committee of Equine Research Institute, Japan Racing Association and Laboratory of Racing Chemistry.

## CONFLICT OF INTERESTS

No competing interests have been declared.

## AUTHOR CONTRIBUTIONS

Yohei Minamijima, Motoi Nomura, Shozo Yamashita, Masayuki Yamada, Shunichi Nagata and Kanichi Kusano contributed to study design, data collection and manuscript preparation. Norihisa Tamura, Hiroshi Mita, Kentaro Fukuda, Atsutoshi Kuwano and Fumio Sato contributed to manuscript preparation. Pierre‐Louis Toutain contributed to pharmacokinetic analysis and manuscript preparation.

## INFORMED CONSENT

Not applicable.

### PEER REVIEW

The peer review history for this article is available at https://publons.com/publon/10.1111/evj.13532.

## Supporting information

Summary

## Data Availability

The data that supports the findings of this study are available from the corresponding author upon reasonable request.
