# Supplementary material for: Medication control of flunixin in racing horses: Possible detection times using Monte Carlo simulations
Source: Equine Vet J. 2021 Nov 25;54(5):979–88. doi: 10.1111/evj.13532 (PMC9546317; doi:10.1111/evj.13532)
Supplement: Supplementary file 1 — Summary [file EVJ-54-979-s001.docx]

**Medikamentenkontrolle von Flunixin Meglumin bei Rennpferden: mögliche Nachweiszeiten anhand von Monte-Carlo Simulationen**

*Korrespondenz E-Mail Adresse: [taisuke.kuroda@equinst.go.jp](mailto:taisuke.kuroda@equinst.go.jp)

**Schlüsselwörter:** Pferd, irrelevante Plasma Konzentration, irrelevante Urine Konzentration, Doping, Medikamentenkontrolle

**Zusammenfassung**

**Hintergrund**:

Bei der Kontrolle von Arzneimitteln ist die Wartezeit der Zeitraum in welchem nach der Verabreichung des Medikaments an keinem Wettkampf teilgenommen werden darf. Die Wartezeit wird festgelegt, indem eine Sicherheitszeit zu einer experimentellen Nachweiszeit addiert wird. Es gibt jedoch keine Berichte über statistische Analysen zur Bestimmung der Nachweiszeit bei Pferden, die mit Flunixin Meglumin behandelt worden sind.

**Ziele:**

Analyse der Populationspharmakokinetik von Flunixin Meglumin bei Pferden durch Erstellung eines Datensatzes für die statistische Analyse der Nachweiszeit und Vorhersagen mittels Monte-Carlo Simulation.

**Studiendesign:**

Experimentelle Studie.

**Methoden:**

Die Plasma- und Urinkonzentration des Arzneimittels wurden nach einmaliger intravenöser (i.v.) Verabreichung von Flunixin meglumin 1.1 mg/kg Körpergewicht (KG) bei 10 Pferden und nach mehrfacher Verabreichung von q 24 h über 5 Tage bei 10 Pferden mittels Flüssigkeitschromatographie mit Massenspektrometrie (LC-MS/MS) gemessen. Die Daten wurden mithilfe eines nichtlinearen Modells mit gemischten Effekten und anschliessender Monte-Carlo-Simulation modelliert. Die irrelevante Plasmakonzentration (IPC) und die irrelevante Urinkonzentration (IUC) wurden nach dem Toutain-Ansatz berechnet. Die Nachweiszeiten wurden unter Berücksichtigung der Zeit nach der letzten Verabreichung für ausgewählte Quantile von 5000 hypothetischen Pferden unter dem von der Internationalen Federation der Rennpferde vorgeschlagenem Screening Limit (ISL) (Plasma: 1 ng/ml, Urin: 100 ng/ml) ermittelt.

**Resultate:**

Bei einer Verabreichung von 1.1 mg/kg KG q 24h lagen die IPC- und IUC-Werte bei 2.0 bzw. 73.0 ng/ml. Die Nachweiszeiten im Plasma über dem ISL für 90 % der simulierten Pferde wurde nach einer einmaligen 1.1 mg/kg Dosis auf 74 Stunden geschätzt. Nach mehreren Verabreichungen über fünf Tage im Abstand von 24, respektive 12 Stunden, wurde die Zeit auf 149, respektive 199 Stunden geschätzt. Die entsprechenden Nachweiszeiten im Urin waren 46, 48 und respektive 104 Stunden.

**Wichtigste Einschränkungen:**

Es wurden nur weibliche Pferde in die Studie aufgenommen.

**Zusammenfassung:**

Die statistischen Nachweiszeiten für verschiedene Flunixin Meglumin Verabreichungen unterhalb des ISLs haben eine Verzögerung der Nachweiszeit im Plasma nach wiederholter Administration.
